# Electrocatalysis of Lindane Using Antimony Oxide Nanoparticles Based-SWCNT/PANI Nanocomposites

**DOI:** 10.3389/fchem.2018.00423

**Published:** 2018-09-21

**Authors:** Kgotla K. Masibi, Omolola E. Fayemi, Abolanle S. Adekunle, El-Sayed M. Sherif, Eno E. Ebenso

**Affiliations:** ^1^Department of Chemistry, Faculty of Natural and Agricultural Sciences, North-West University, Mmabatho, South Africa; ^2^Material Science Innovation and Modelling Research Focus Area, Faculty of Natural and Agricultural Sciences, North-West University, Mmabatho, South Africa; ^3^Department of Chemistry, Obafemi Awolowo University, Ile-Ife, Nigeria; ^4^Center of Excellence for Research in Engineering Materials, King Saud University, Al-Riyadh, Saudi Arabia; ^5^Electrochemistry and Corrosion Laboratory, Department of Physical Chemistry, National Research Centre, Cairo, Egypt

**Keywords:** polyaniline, single-walled carbon nanotubes, antimony oxides nanoparticles, lindane, square-wave voltammetry, cyclic voltammetry

## Abstract

This work describes the chemical synthesis of antimony oxide nanoparticles (AONPs), polyaniline (PANI), acid functionalized single-walled carbon nanotubes (fSWCNTs), and the nanocomposite (AONP-PANI-SWCNT) as catalyst for the trace detection of lindane. Successful synthesis of the nanomaterials was confirmed by Fourier transform infrared (FT-IR) spectroscopy, ultraviolet-visible (UV-Vis) spectroscopy, x-ray diffraction (XRD) spectroscopy, and scanning electron microscopy (SEM). Cyclic voltammetry (CV) and electrochemical impedance spectroscopy (EIS) were used for investigating the electrochemical behavior of the modified electrodes in the ferrocyanide/ferricyanide ([Fe(CN)_6_]^4−^/[Fe(CN)_6_]^3−^) redox probe. GCE-AONP-PANI-SWCNT exhibited faster electron transport properties as well as higher electroactivity as compared to bare-GCE, GCE-AONPs, GCE-PANI, and GCE-SWCNT electrodes. Electrocatalytic studies further showed that GCE-AONP-PANI-SWCNT modified electrode was stable (after 20 scans) with only a small current drop in lindane (0.57%). The GCE-AONP-PANI-SWCNT electrode with low detection limit of 2.01 nM performed better toward the detection of lindane as compared to other studies in literature. The GCE-AONP-PANI-SWCNT electrode is highly selective toward the detection of lindane in the presence of various organic and inorganic interfering species. Real sample analysis of river water and tap water samples using the developed sensor gave satisfactory percentage recoveries therefore confirming the potential of the proposed sensor for practical application.

## Introduction

Organochlorine pesticides (OCPs) are chlorinated hydrocarbons emanating from persistent organic pollutants (POPs) used extensively from early 1900 in agriculture and mosquito control. Like POPs they accumulate in the environment and are very persistent (Jones and de Voogt, 1999; Chrysikou et al., 2008; El-Shahawi et al., 2010; Van Dyk and Pletschke, 2011; Salihovic et al., 2012; Gill et al., 2013). Examples of these POPS are dichlorodiphenyltrichloroethane, dieldrin, mirex, kepone, lindane, alanchlor, methoxychlor, endosulfan, chlordane, dicofol, heptachlor, toxaphene, and benzene hexachloride (Jones and de Voogt, 1999; Abdollahi et al., 2004; Chrysikou et al., 2008; El-Shahawi et al., 2010; Van Dyk and Pletschke, 2011). Lindane (γ-hexachlorocyclohexane (γ-HCH)) is an organochlorine pesticide with properties such as molecular formula C_6_H_6_Cl_6_, molecular weight of 290.85 g/mol, and melting point of 112–113°C. Physically, lindane appears as a colorless-to-white powder with a musty odor (Abdollahi et al., 2004; Prathap and Srivastava, 2013; Anirudhan and Alexander, 2015). Lindane was globally banned for agricultural use, however the pesticide is still widely used in the production of shampoos, lotions to treat head lice, and scabies (Blair et al., 1998; Prathap and Srivastava, 2013). The accumulation of lindane has been detected globally in human breast milk, human blood, and adipose tissue (Prathap and Srivastava, 2013), which have resulted to a number of adverse human health effects. These health effects include neurological disorders such as seizures, convulsions, vertigo as well as the elevated risks of liver and breast cancer. Most of the reported health effects have been related to agricultural use and chronic occupational exposures (Blair et al., 1998; Wilson and Tisdell, 2001; Prathap and Srivastava, 2013; Anirudhan and Alexander, 2015).

Sophisticated analytical techniques have been reported for quantitative detection of these pesticides. These methods include; gas chromatography (Sen et al., 2011), gas chromatographic-mass spectrometry (Rodrigues et al., 2007), colorimetric methods (Lichtenstein et al., 1956), thin layer chromatography (Norén and Westöö, 1968), and high-performance liquid chromatography (Vassilakis et al., 1998). The analytical techniques are known to be very expensive and complex, requiring highly skilled personnel and time consuming sample preparations, they are also not suitable for onsite monitoring (Prathap et al., 2015, 2016; Fayemi et al., 2016). Therefore, electrochemical sensors are emerging as a technology of choice for the electrochemical detection of important biological and environmental analytes (Fayemi et al., 2016; Prathap et al., 2016; Zhuang et al., 2016). Electrochemical sensors are known to possess a vast number of advantages which include high sensitivity, rapid response, convenience in operation, and low fabrication costs (Prathap et al., 2016; Zhuanga et al., 2017; Chena et al., 2018). Different materials have been employed for the fabrication of these sensors and an example of such materials is carbon nanotubes. Carbon nanotubes (CNTs) possess unique properties such as high surface area to volume ratio, tensile strength, chemical stability, good electrical conductivity, and high adsorption capacity. CNTs have evolved as a good supporting material for surface modification of electrodes (Vairavapandian et al., 2008; Adekunle et al., 2010a, 2011b; Cesariono et al., 2013). There are reports on the use of single-walled carbon nanotubes (SWCNTs) as a supporting material for surface modification of electrodes in electrochemical studies of some important analytes. Literature studies include nitrite electrochemical sensor based on Prussian blue/single-walled carbon nanotubes modified pyrolytic graphite electrode (Adekunle et al., 2011b), electrocatalytic detection of dopamine at single-walled carbon nanotubes functionalized with iron(III)oxide nanoparticles (Adekunle et al., 2010a) and probing the electrochemical behavior of SWCNT–cobalt nanoparticles and their electrocatalytic activities toward the detection of nitrite at acidic and physiological pH conditions (Adekunle et al., 2010b). Conducting polymers such as polyaniline (PANI) are of great importance due to their unique properties such as good environmental stability, ease of synthesis, oxidation or protonation, adjustable electro-optical properties (Yu et al., 2008; Tovide et al., 2014), and wide application potential (Trchová and Stejskal, 2011). PANI is known to have found potential applications in various areas like batteries (Dalui et al., 2008), separation devices (Sairam et al., 2006), light-emitting diodes (Prathap and Srivasta, 2011), electrorheological material (Zhang et al., 2002) and molecular sensors (Rahman et al., 2008). There is currently an interest in the use of metal oxide-CNTs nanocomposites, and metal oxide-polymer composites in electrochemistry to improve the performance of electrochemical detection of biological and environmental analytes. Also, owing to their intrinsic properties such as negative overpotential to hydrogen evolution, large scan potential range, antimony (Sb) based electrodes have received growing attention in electrochemistry (Prathap and Srivasta, 2011; Tovide et al., 2014). Despite these characteristics there are few number of reports describing the use of electrodes based on antimony and its oxides.

Thus, the novelty in this work is to explore the synergistic electrical properties and sensitivity of a nanocomposite based electrode materials made from carbon nanotubes (CNT), polyaniline (PANI) and antimony oxide (AO) toward nano detection of lindane and related pesticides in river and tap water samples. To the best of our knowledge, this study represents the first time a highly selective GCE-AONP-PANI-SWCNT electrode architecture for lindane detection is described. The study also provides information on the mechanism of electron transport between the surface active material and the underlying electrode or the analyte in solution for enhanced electrochemical response. This study therefore describes successful synthesis of GCE-AONPS-PANI-SWCNT electrode and its application for the electrochemical detection of lindane in river and tap water samples.

## Methods

### Materials, reagents, and instruments

Lindane (C_6_H_6_Cl_6)_, pristine single-walled carbon nanotubes (SWCNT; 90% purity, 0.7–1.1 nm), tetrabutylammonium bromide (TBAB), and N,N-Dimethylformamide (DMF) were purchased from Sigma-Aldrich. Methanol, nitric acid (HNO_3_), sulphuric acid (H_2_SO_4_), antimony chloride (SbCl_3_), polyvinyl alcohol (PVA), sodium hydroxide (NaOH), hydrochloric acid (HCl), aniline (99%), ammonium peroxodisulfate (APS) [(NH_4_)_2_S_2_O_8_], sodium phosphate monobasic (NaH_2_PO_4_), sodium phosphate dibasic (Na_2_HPO_4_) and ethanol were of analytical grade and obtained from Merck chemicals. A 3 mm glassy carbon electrode (GCE) was used as the working electrode. Alumina micro powder (0.3 μm alumina slurries) and polishing pads were used for polishing the working electrode. Phosphate buffer solution (PBS) of 0.1 M at pH 7 was prepared by using suitable amounts of NaH_2_PO_4_ and Na_2_HPO_4_ and then adjusted with NaOH. During electrochemical experiment purging of the prepared solutions was done by using nitrogen for oxygen elimination and also to prevent any form of oxidation. The electrochemical workstation used is the Autolab Potentiostat PGSTAT 302 with GPES software version 4.9. UV-vis and FTIR characterization were carried out using Agilent Technology, UV-1901, and Cary 600 series FTIR spectrometer, USA, respectively. Scanning electron microscope and X-ray diffraction spectrophotometer were used for morphological characterization of the nanomaterials.

### Synthesis of antimony oxide nanoparticles (AONPs) and polyaniline (PANI)

About 228 mg of SbCl_3_ was dissolved in 100 mL of 1 M HCl, then 3 g of PVA was added. The mixture was dispersed by bath sonication for 15 min. Approximately 12 mL of 10 M NaOH was added slowly to the mixture until the mixture color changed to transparent-pale yellow. The mixture was then refluxed for 1 h and the color became more intense. The mixture was heated at 350°C for another 1 h to obtain a dry powder samples (Zhang et al., 2001). PANI was synthesized using a reported method by Kavitha et al. (2013). Quantitative 50 mL of 1 M HCl and 2 mL aniline were stirred together in 250 mL flask on a magnetic stirrer. Concurrently, 50 mL of 1 M HCl and 5 g of APS aqueous solution were also stirred together. This solution was added dropwise to the former solution and polymerized at a temperature of 70°C for 10 h to complete the reaction. The obtained precipitate was filtered and washed with 1 M HCl followed by distilled water until a clear filtrate was achieved. The final residue was dried for 24 h in the oven at 60°C.

### Acid treatment of SWCNT

About 40 mg pristine SWCNTs were first dispersed in 40 mL DMF for 40 min at 40°C. The resulting dispersion was then filtered and washed with 10 mL methanol followed by deionized water. The solid was transferred into a glass vial containing 20 mL of 8 M HNO_3_ and sonicated for 30 min at 40°C followed by the addition of 40 mL deionized water. After filtration the recovered solid was washed with deionized water until the filtrate was neutral and finally washed with 10 mL of methanol and 10 mL of DMF. Lastly the solid was dispersed in 20 mL DMF by bath sonication for 60 min at 40°C, it was filtered and dried to get powder form of fSWCNTs (Tchoul et al., 2007).

### Preparation of nanocomposites

Approximately 2 mg of fSWCNT suspended in 2 mL DMF was mixed with 2 mg each of PANI and AONPs. The mixture was stirred for 48 h at room temperature. The formed AONP-PANI-SWCNT nanocomposites was dried at 25°C overnight for the solvent to evaporate (Fayemi et al., 2015).

### Electrode modification and characterization of the electrode

The electrode was modified by the drop-cast method (Adekunle et al., 2011a; Silwana et al., 2015). GCE surface was cleaned first by a gentle polish with 0.3 μm alumina slurries on a polishing pad followed by a gentle rinse with distilled water. The electrode was then sonicated in ethanol for 5 min and again in distilled water for another 5 min. About 5 mg each of the prepared nanomaterials, AONPs, PANI, fSWCNTs, and AONP-PANI-SWCNT were suspended in 1 mL DMF. Each suspension was then dispersed by ultrasonic vibration for 30 min, and a 20 μL aliquot of this dispersion was dropped onto the GCE surface and dried at 50°C to obtain the modified electrodes. Fourier transform infrared spectroscopy (FT-IR), ultraviolet-visible (UV-Vis) spectrophotometry, X-ray diffraction (XRD) spectroscopy, and scanning electron microscopy (SEM) were used for the structural and morphological characterization of the synthesized nanomaterials.

### Electrochemical studies

Electrochemical experiments were carried out to establish the successful modification of the electrodes, electron transport and electrocatalytic properties of the bare and modified GCE. Bare or modified glassy carbon electrode (GCE) disk (*d* = 3.0 mm in Teflon) was used as the working electrode, platinum disk as counter electrode and Ag/AgCl, KCl (sat'd) as reference electrode. A bench top pH/ISE ORION meter, model 420A, was used for pH measurements. All solutions were de-aerated by bubbling with nitrogen prior to each electrochemical experiment. Experiments were performed at 25 ± 1°C. Electrochemical experiments were carried out using an AUTOLAB Potentiostat PGSTAT 302 (Eco Chemie, Utrecht, and The Netherlands) driven by the GPES software version 4.9. Electrochemical impedance spectroscopy (EIS) measurements were performed with an AUTOLAB Frequency Response Analyser (FRA) software between 0.1 Hz and 100 kHz using a 5 mV rms sinusoidal modulation with a solution of 5 mM mixture of K_4_Fe(CN)_6_ and K_3_Fe(CN)_6_ (1:1) prepared with pH 7.0 phosphate buffer solution (PBS). CV experiments were carried out by running the bare and modified GCE in 0.1 M buffer solution (PBS, pH 7.0) alone, 5 mM [Fe(CN)_6_]^4−/3−^ solution prepared in 0.1 M PBS and 9 μM of lindane prepared in 60:40 methanol/water containing 0.05 M TBAB as supporting electrolyte.

## Results and discussion

### Characterization of synthesized nanomaterials

#### Fourier transform infrared spectroscopy

**Figure 1:**
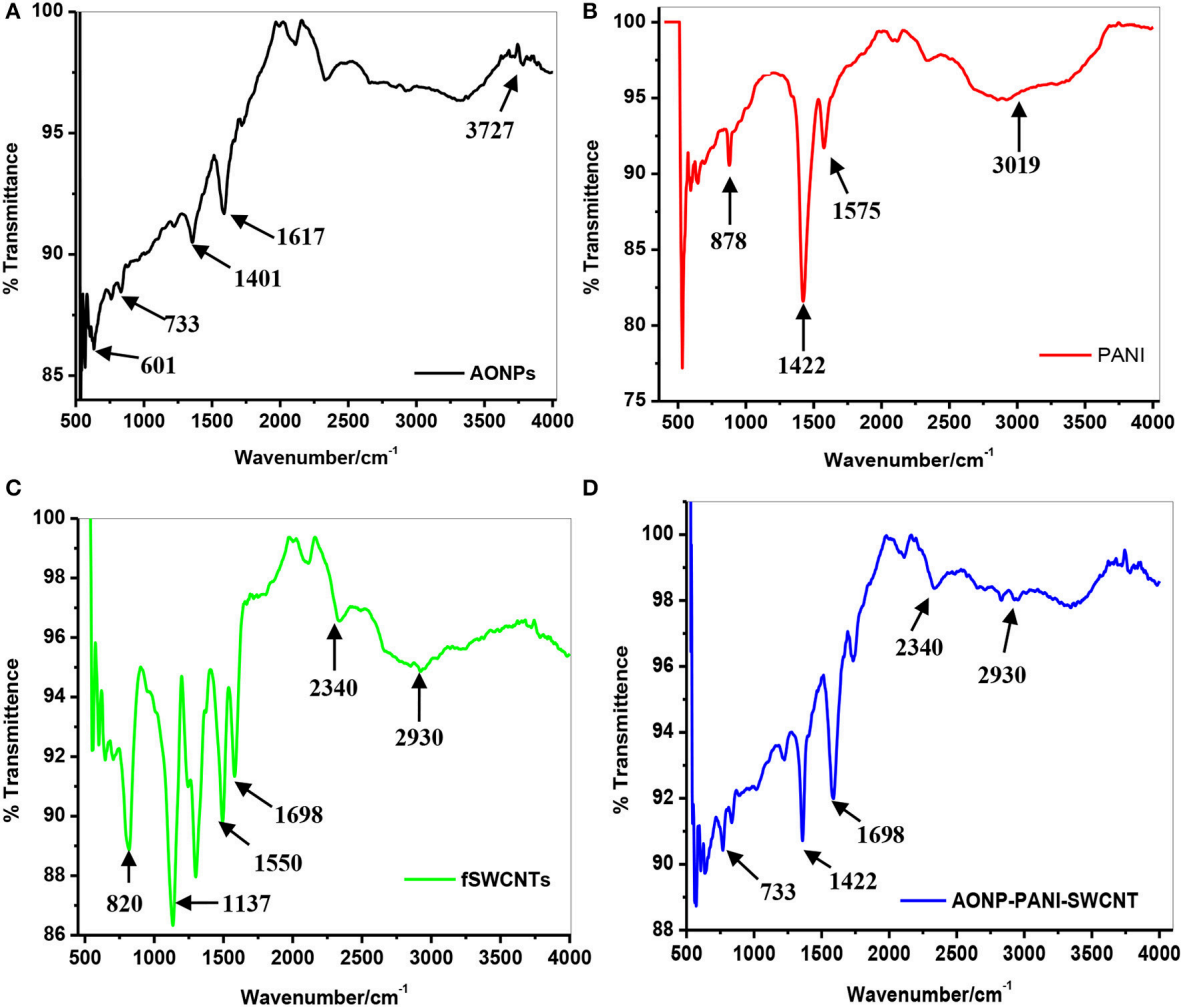
FT-IR spectra of **(A)** AONPs, **(B)** PANI, **(C)** fSWCNTs, and **(D)** AONP/PANI/SWCNT.

Figure 1 depicts comparative FT-IR spectra of (a) AONPs, (b) PANI, (c) fSWCNTs and (d) AONP-PANI-SWCNT. From the AONPs spectrum (Figure 1A) the peak at 3727 cm^−1^ was assigned to the stretching and bending vibrations of OH group in water. The peak at 733 cm^−1^ was assigned to the Sb-O-Sb vibrations, whilst the small absorption band at 601 cm^−1^ was attributed to the metal oxygen stretching vibration (Zhang et al., 2001; Kaviyarasu et al., 2013; Silwana et al., 2015). The PANI spectrum (Figure 1B) shows characteristic peaks at ~878, 1422, 1575, and 3019 cm^−1^, respectively. The sharp peaks observed at 1575 and 1422 cm^−1^ were due to the C = N and C = C stretching vibrations of quinoid and benzenoid rings, respectively (Cesarino et al., 2011; Konyushenko et al., 2011; Abdolahi et al., 2012; Olad et al., 2012; Kavitha et al., 2013; Prathap et al., 2013). The peak at 878 cm^−1^ can be assigned to the C-H out of plane bending (Abdolahi et al., 2012; Olad et al., 2012; Prathap et al., 2013). The broad peak at ~3019 cm^−1^ represents the N-H stretching vibrations (Olad et al., 2012). Figure 1C depicts FT-IR spectrum of acid functionalized single-walled carbon nanotubes and from the spectrum, characteristic peaks were observed at 2930, 2340, 1698, 1104, and 820 cm^−1^. The peaks at 2930 and 2340 cm^−1^ observed in the high frequency region were attributed to the symmetric mode and anti-symmetric mode of CH_2_ (Tchoul et al., 2007; Helali et al., 2016). The peak observed at 1698 cm^−1^ was assigned to the carbonyl stretching vibration demonstrating the introduction of a carboxyl group on the surface of the nanotubes (Xu et al., 2015). The peak at 1104 cm^−1^ was assigned to either the SWCNT defects or residual carbon impurities or both (Helali et al., 2016).

Figure 1D shows the spectrum of the nanocomposite AONP-PANI-SWCNT with characteristic peaks of the various nanoparticles used to synthesize the nanocomposite. The peak at 733 cm^−1^ attributed to the Sb-O-Sb stretching vibration confirms the presence of AONPs in the nanocomposite (Zhang et al., 2001; Kaviyarasu et al., 2013), while the C = C stretching vibrations of benzoid rings were represented by the peak at 1422 cm^−1^. The peak observed at 1698 cm^−1^ was assigned to the carbonyl stretching vibration and the peaks at 2930 and 2340 cm^−1^ were due to the symmetric and anti-symmetric mode of CH_2_ (Helali et al., 2016).

#### Ultraviolet-visible spectroscopy

**Figure 2:**
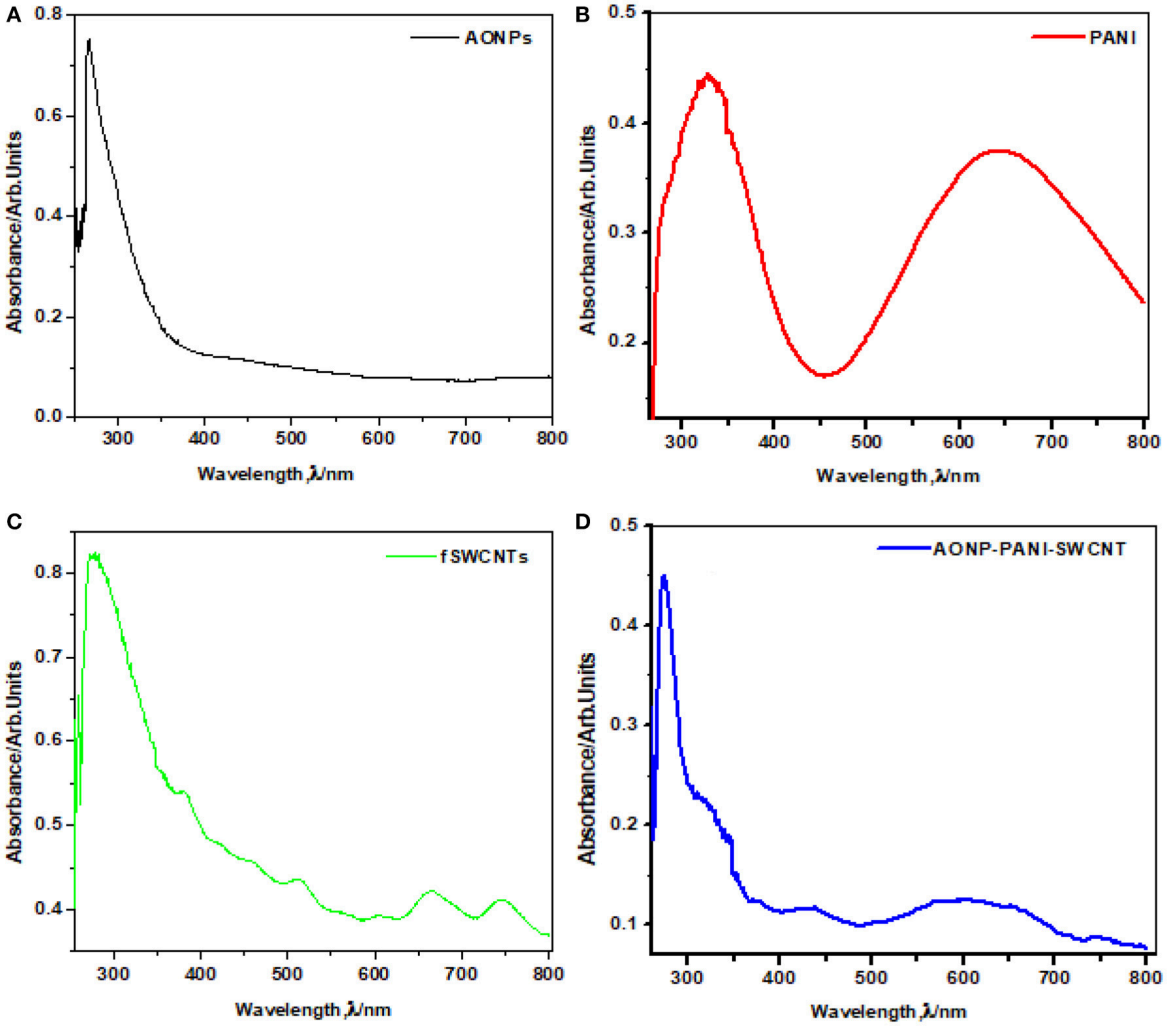
UV-vis spectra of **(A)** AONPs, **(B)** PANI, **(C)** fSWCNTs, and **(D)** AONP-PANI-SWCNT.

Figure 2 shows the comparative UV-vis spectra of (a) AONPs, (b) PANI, (c) fSWCNTs and (d) AONP-PANI-SWCNTs. AONPs spectrum. A sharp peak at about 260 nm is characteristic of antimony oxide nanoparticles formation Figure 2D (Khalef, 2013). In Figure 2B peaks at 341 nm was attributed to the π-π^*^benzenoid transition while 650 nm was assigned to the quinoid transition in PANI (Konyushenko et al., 2011; Prathap and Srivasta, 2011). The absorption spectrum of the fSWCNTs (Figure 2C) showed a significant loss of the van Hove band structure typical for pristine SWCNTs and usually centered around 650 nm and 900 nm (Chiang et al., 2001; Peng et al., 2003; Price and Tour, 2006). Figure 2D shows the spectrum of AONP-PANI-SWCNTs composite with characteristic peaks of the AONPs at around 260 nm, and quinoid transition peak of PANI around 650 nm, which are indicative of the presence of AONPs and PANI in the nanocomposite.

#### X-ray diffraction spectroscopy

**Figure 3:**
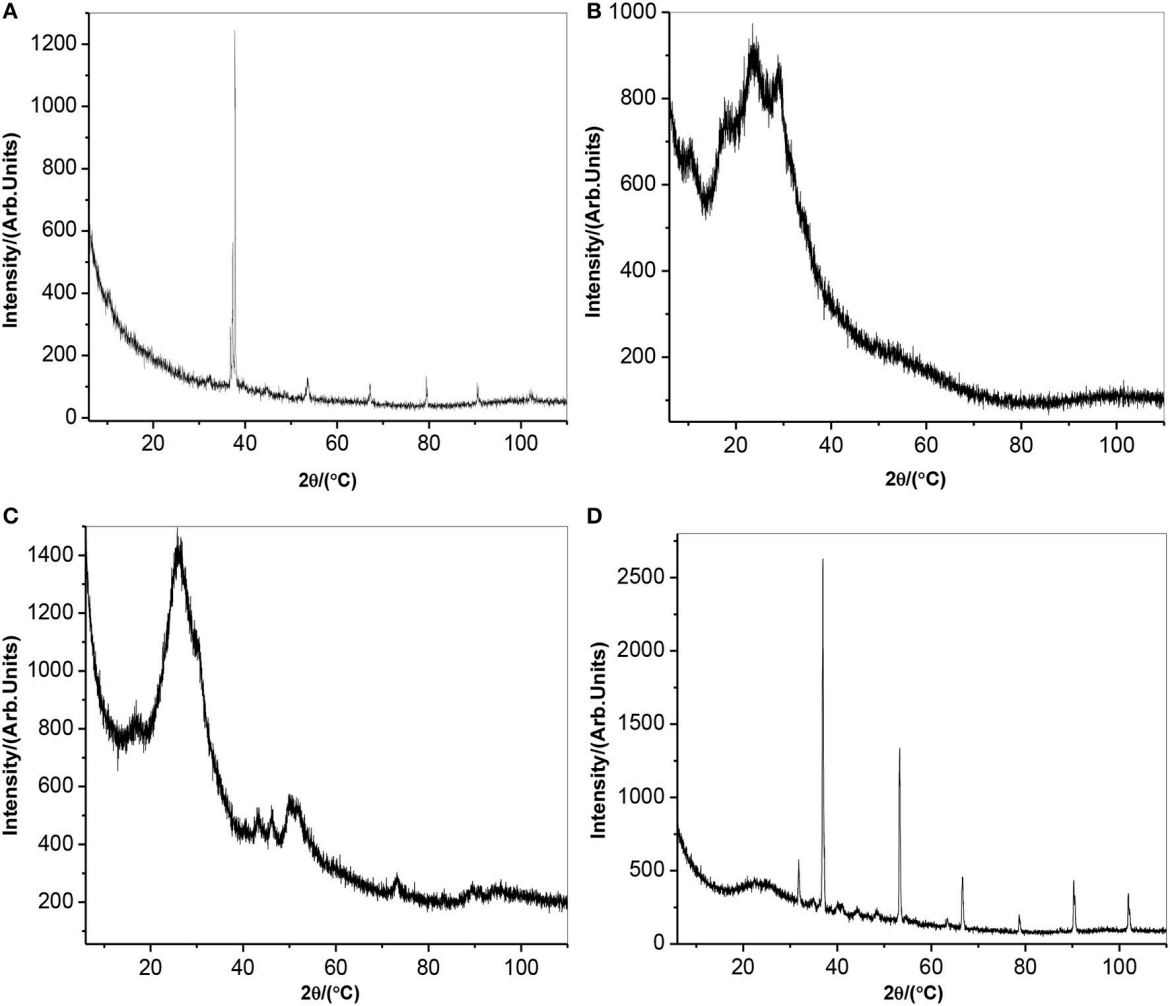
XRD spectra of **(A)** AONPs, **(B)** PANI, **(C)** fSWCNTs, and **(D)** AONP-PANI-SWCNT.

Figure 3 depicts x-ray diffraction pattern of (a) AONPs, (b) PANI, (c) fSWCNTs, and (d) AONP-PANI-SWCNT. Antimony oxide nanoparticles are generally known to exist in a wide range of compositions and are also known to display interconvertible polymorphism. The two most common forms are cubic and orthorhombic consisting of Sb_4_O_6_ molecules and chains of SbO_3_ trigonal pyramids (Khalef, 2013). From the AONPs spectrum (Figure 3A), the sharp and intense peak observed at 2Θ = 37.5° corresponds to the (2 0 0) plane indicating preferred crystallographic orientation similar to the sample prepared in iso-propanol (Naidu et al., 2009). Similar increase in intensity for (1 1 0) and (2 0 0) was also reported (Deng et al., 2006), confirming successful oxidation of Sb metal to form nano-rods like Sb_2_O_3_. From the literature it was reported that PANI prepared at pH 7 exhibited high crystallinity with the XRD pattern showing an intense peak at 2Θ = 6.4,^31^ as shown in the XRD pattern obtained for PANI in Figure 3B. Other peaks observed were at 2Θ = 18.5°, 19.8°, 23.4°, 25.5°, 26.4°, and 28.4° confirming the high order structure of PANI, however broaden peaks at 20.4° and 25.5° suggests semi-crystallinity and amorphous nature of the PANI with an average particle size of about 3.8 nm (Prathap and Srivasta, 2011). The XRD spectrum of fSWCNTs is depicted by Figure 3C and a broad peak at 2Θ = 28° was observed. The peak is a typical pattern of an amorphous structure (Ng et al., 2005), numerous other x-ray studies on carbon materials also suggested that the peak is due to the (0 0 2) plane of the turbo-stratic graphitic layer arising from the acid treatment degradation of SWNTs. The peak at 2Θ = 43° was attributed to the presence of (1 0 0) peak in graphite. The XRD spectrum of AONP-PANI-SWCNT composite is shown in Figure 3D, the intense peak at 2Θ = 37.5° corresponding to the (2 0 0) plane of AONPs confirms composite formation with well crystallized AONPs. The broad peak at 2Θ = 20.4° was attributed to the amorphous structure of fSWCNTs as well as semi-crystallinity of PANI.

#### Scanning electron microscopy

**Figure 4:**
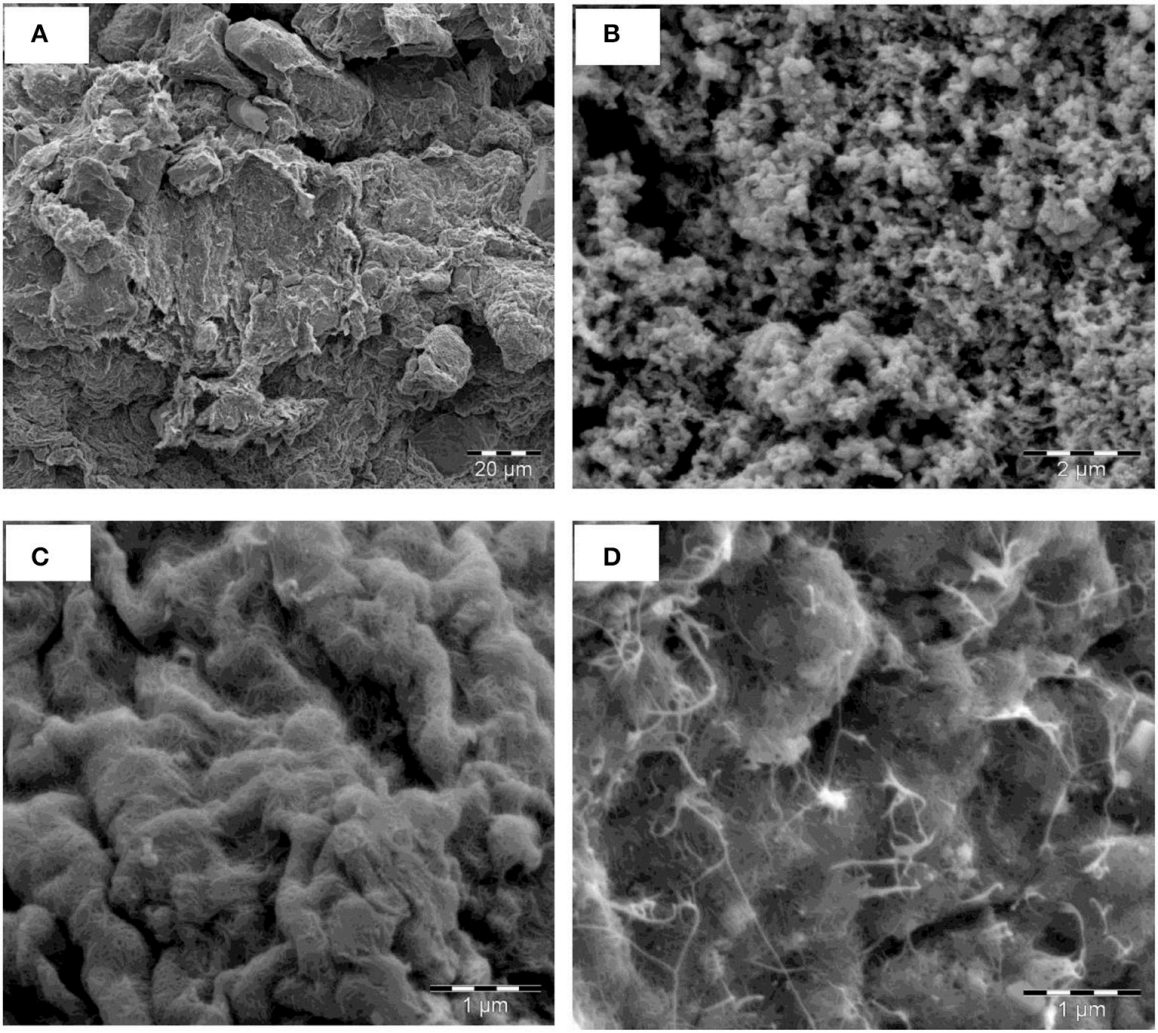
SEM images of **(A)** AONPs, **(B)** PANI, **(C)** fSWCNTs and **(D)** AONP-PANI-SWCNT.

The morphology of the synthesized nanomaterials were depicted by their respective SEM images. Figure 4 shows the SEM images of (a) AONPs, (b) PANI, (c) fSWCNTs and (d) AONP-PANI-SWCNT. The SEM image in Figure 4A showed that the AONPs appeared unevenly distributed and clustered randomly; the image also depicts compacted flaky platelets. The SEM image for PANI (Figure 4B) showed porous morphology with evenly distributed agglomerations. Figure 4C represents the SEM image for fSWCNTs showing unevenly distributed and tangled porous tube-like structures. The SEM image for the composite AONP-PANI-SWCNT (Figure 4D) shows a characteristic morphology for antimony oxide nanoparticles with random clusters but here the clusters are tangled by the SWCNTs resembling a web like assembly.

### Electrochemical characterization

#### Cyclic voltammetric experiments

**Figure 5:**
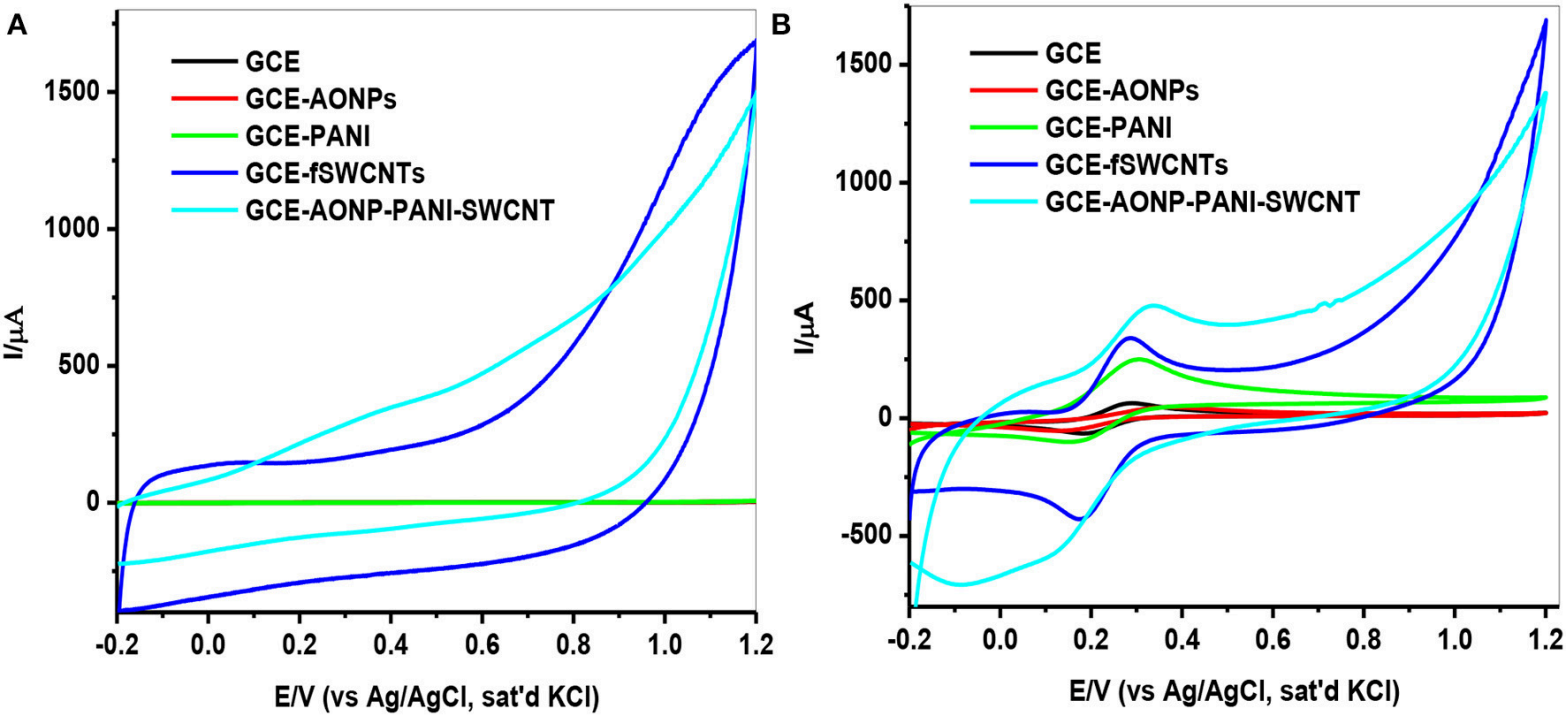
CVs of modified electrodes in **(A)** 0.1 M PBS solution **(B)** 5 mM [Fe(CN)_6_]^3−/4−^ solution at a scan rate of 25 mVs^−1^.

Investigation of the electrochemical properties of the bare and modified electrodes were carried out by using cyclic voltammetry in 0.1 M PBS (pH 7.0), then in 5 mM [Fe(CN)_6_]^3−/4−^ solution at a scan rate of 25 mVs^−1^. Figure 5A represents the comparative CVs for the modified electrodes in buffer solution. From the voltammograms, it was observed that the electrodes GCE-fSWCNTs and GCE-AONPs-PANI-SWCNT exhibited higher current responses. This may be due to the influence of SWCNTs which are known to enhance the electronic communication between the electrode and the electrolyte solution, thereby acting as “Coulombic Islands” (electron transfer stations) and tunneling electrons between the electrode surface and redox species (Liu et al., 2012). A similar electrochemical behavior were further observed in the [Fe(CN)_6_]^3−/4−^ solution. As shown in Figure 5B, the cyclic voltammetric responses of the electrodes exhibited a pair of well-defined redox peaks characteristic of one-electron transfer redox process in the region −0.2–0.4 V typical of [Fe(CN)_6_]^3−/4−^ redox process (Adekunle et al., 2015). The surface coverage (Γ) (Shap et al., 1979; Fu et al., 2009) for each electrode was calculated to be 65.8, 53.3, 34.9, 6.45, and 8.80 nmol.cm^−2^ for GCE-AONP-PANI-SWCNT, GCE-fSWCNTs, GCE-PANI, GCE-AONPs, and bare GCE, respectively.

Table 1 summarizes the electrochemical data and various electrochemical parameters obtained for the electrodes in 5 mM [Fe(CN)_6_]^3−/4−^ redox probe such as ratio of anodic and cathodic peak current (I_pa_/I_pc_), surface coverage (Γ/mol.cm^−2^) and peak-to-peak separation (ΔE_p_/V). The I_pa_/I_pc_ values obtained are closer to 1 indicating that the couple is reversible (Bard and Faulkner, 2001), and the peak-to-peak separation (ΔE_p_) values indicate that the electron transport was fastest at the GCE-fSWCNTs electrode (90.3 mV/s) and slowest at the GCE-AONPs electrode (178.3 mV/s). The overpotential was relatively small at GCE-AONP-PANI-SWCNT nanocomposite electrode because the higher current response recorded at the electrode (anode: 451 μA; cathode: 499 μA), was greater than that at GCE-fSWCNTs (anode: 365 μA; cathode: 422 μA).

**Table 1:** Summary of cyclic voltammetric parameters obtained for bare and modified electrodes in 5 mM [Fe(CN)_6_]^4−^/[Fe(CN)_6_]^3−^ solution prepared in 0.1 M PBS.

**Electrode**	**I_pa_ /μA**	**I_pc_/μA**	**I_pa_/ I_pc_**	**Γ/ mol.cm^−2^**	**E_pa_/mV**	**E_pc_/mV**	**ΔEp/mV**
GCE	60.4	61.7	0.98	8.80	271.1	161.3	109.8
GCE-AONPs	44.2	49.9	0.89	6.45	337.1	158.8	178.3
GCE-PANI	239	199	1.20	34.9	278.5	161.3	117.2
GCE-fSWCNTs	365	422	0.86	53.3	251.6	161.3	90.3
GCE-AONP- PANI-SWCNT	451	499	0.90	65.8	278.5	161.3	117.2

### Electrochemical impedance spectroscopy

**Figure 6:**
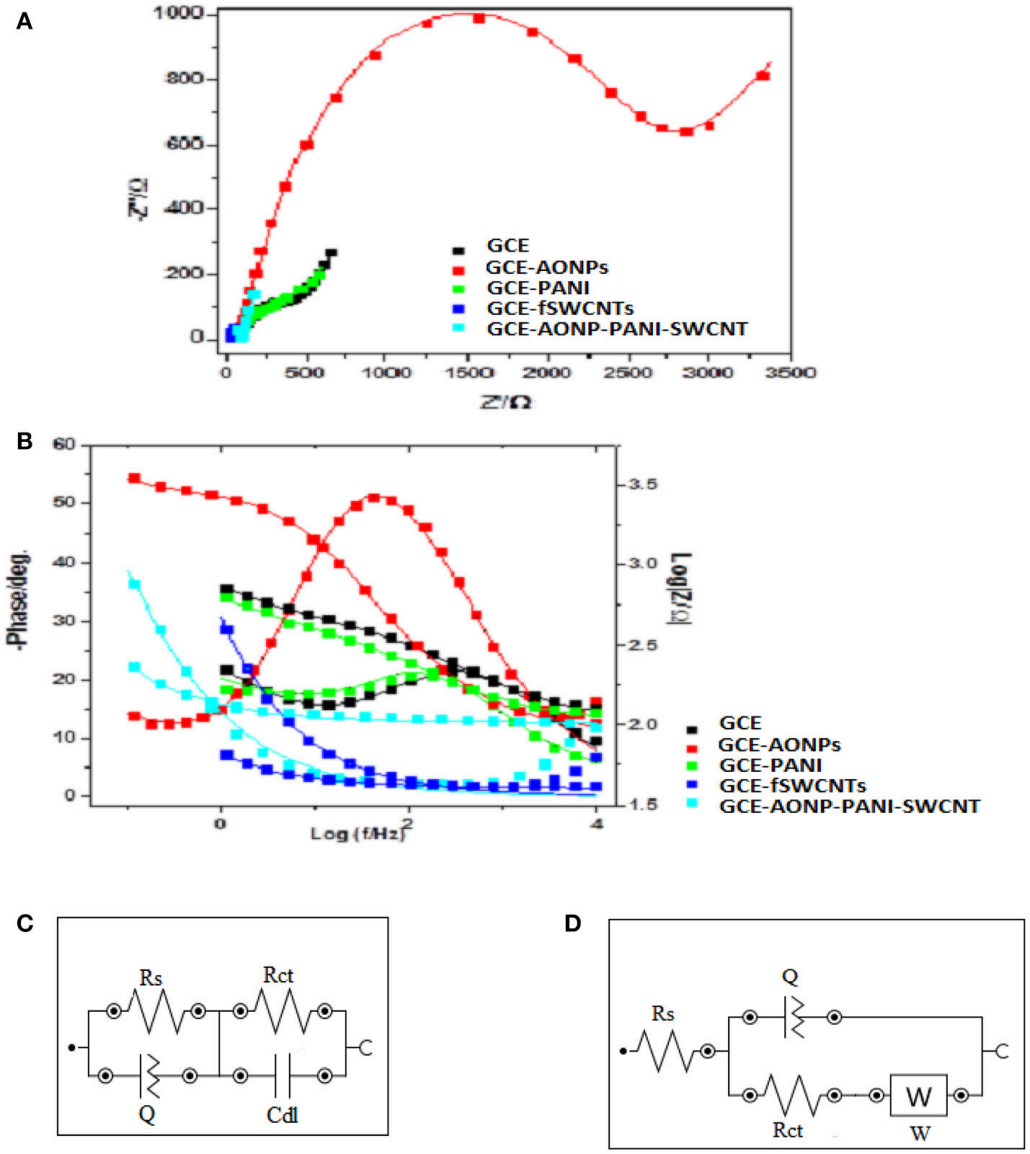
**(A)** Nyquist plots for the modified electrodes in 5 mM FECN solution at a fixed potential of 0.28 V and **(B)** Bode plots for the modified electrodes in 5 mM [Fe(CN)_6_]^4−^/[Fe(CN)_6_]^3−^ solution showing the plots of—phase angle/deg. vs. log(f/Hz) and log|Z/Ω| vs. log(f/Hz). **(C,D)** are the Randle's equivalent circuits used for fitting the EIS data for GCE, GCE-AONPs, GCE-PANI, GCE-fSWCNTs, and GCE-AONP-PANI-SWCNT electrodes, respectively.

EIS was carried out to investigate the electron transfer properties of the various electrodes in [Fe(CN)_6_]^4−^/[Fe(CN)_6_]^3−^ solution. The experiments were conducted at frequency range of 0.1–100 kHz. Figure 6 depicts the Nyquist plots (a) and Bode plots (b) obtained for the bare-GCE, GCE-AONPs, GCE-PANI, GCE-fSWCNTs, and GCE-AONP-PANI-SWCNT electrodes, whilst Figures 6C,D represent the circuit model used to fit the EIS data. The circuit represented by Figure 6C was used to fit the data of the bare-GCE, GCE-AONPs and GCE-PANI and circuit represented by Figure 6D was used to fit the data for GCE-SWCNTs and GCE-AONP-PANI-SWCNT. The circuits parameters were described as R_s_, R_ct_, C_dl_, and W representing the electrolyte resistance, charge transfer resistance, double layer capacitance, and Warburg impedance, respectively (Lisdat and Shäfer, 2008; Adekunle et al., 2011b).

Table 2 summarizes the EIS data obtained for the various modified electrodes. It was observed from the results that GCE-fSWCNT and GCE-AONP-PANI-SWCNT electrodes had the lowest R_ct_ values (Table 2) than the bare GCE, GCE-AONPs and GCE-PANI indicating that modification particularly with the f-SWCNT on the GCE significantly increased the conductivity of the electrode and enhanced charge transfer.

**Table 2:** Summary of the impedance data obtained for the modified electrodes in 5 mM [Fe(CN)_6_]^3−/4−^.

**Electrode**	**R_s_ (Ω cm^2^)**	**R_ct_ (Ω cm^2^)**	**Q (× 10^−3^)**	**W (Ω cm^2^) (x10^−3^)**	**C_dl_ (nF)**	**Chi-square (× 10^−3^)**
GCE	108.3 (0.456)	362 (0.76)	0.0497 (3.508)	1.616 (0.822)	–	2.667
GCE-PANI	106.5 (1.543)	371 (5.138)	0.1103 (11.93)	1.38 (4.632)	–	34.116
GCE-AONPs	97.5 (1.746)	2740 (2.439)	0.0226 (1.069)	1.344 (11.746)	–	123.64
GCE-fSWCNTs	15.87 (0.0)	25.01 (0.934)	7.689 (1.782)	–	125.7 (0.406)	9.5302
GCE-AONP-PANI -SWCNT	60.7 (7.85)	46.6 (9.563)	7.79 (1.181)	–	271.6 (0.521)	32.658

*The values in parenthesis represent the percentage error of the data fitting*.

### Electrocatalytic studies of lindane

#### Electrocatalytic reduction of lindane

**Figure 7:**
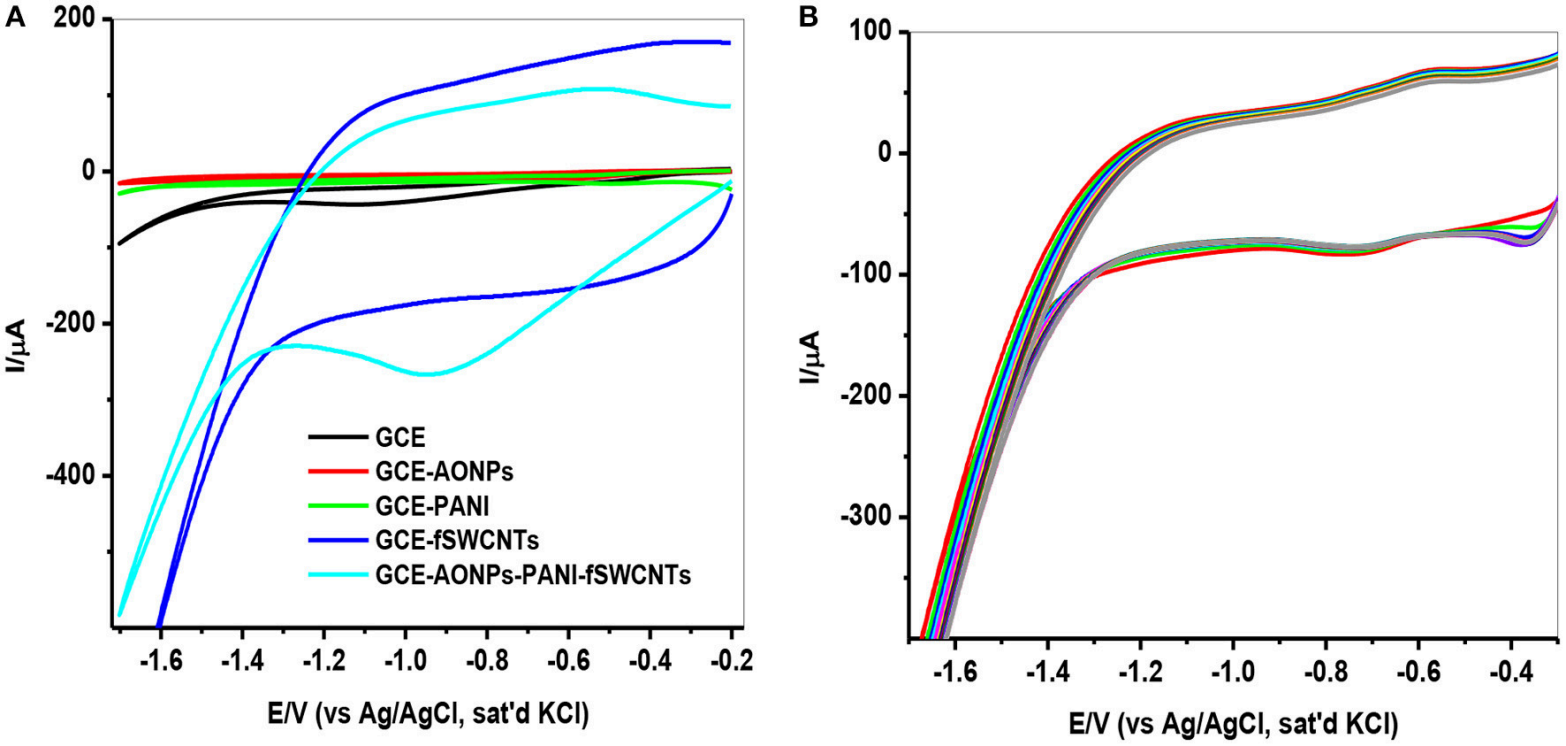
**(A)** CVs of the GCE modified electrodes in the presence of 9 μM lindane at a scan rate of 25 mVs^−1^. **(B)** CVs (20 scans) of GCE-AONP-PANI-SWCNT modified electrode in the presence of 9 μM lindane at a scan rate of 25 mVs^−1^.

The electrochemical behavior of 9 μM lindane at a potential of −0.2 to −1.7 V and a scan rate of 25 mVs^−1^ using CV technique is represented in Figure 7. Figure 7A shows the comparative cyclic voltammograms obtained for GCE, GCE-AONPs, GCE-PANI, GCE-fSWCNTs, and GCE-AONP-PANI-SWCNT electrodes. The obtained reduction peaks with no anodic peaks suggests that the electrocatalysis of lindane is an irreversible reaction and similar results have been reported (Kumaravel et al., 2013; Kaur et al., 2015; Fayemi et al., 2016; Otles, 2016). From the voltammogram in Figure 7A, the reduction potential for lindane was observed at −1.14, −1.00, −0.93, and −0.88 V at the bare GCE, GCE-PANI, GCE-AONP, and GCE-AONP-PANI-SWCNT electrodes, respectively. There was no observable peak for lindane at the GCE-fSWCNT modified electrode probably due to the capacitive current of the electrode. However, the introduction of the AONP in GCE-AONP-PANI-SWCNT electrode increased the electrode conductivity and catalysis with well pronounced and observable lindane reduction peak as compared to SWCNT or PANI modified electrodes alone. Generally, the modified electrodes shifted to a more positive potential as compared to that of the bare GCE. It was interesting to note that the reduction current for GCE-AONP-PANI-SWCNT composite modified electrode significantly increased by over 6-folds as compared to that of the bare GCE. This may be due to the high electrocatalytic activity and strong adsorption capabilities of the nanomaterial on the modified electrodes and its effective interaction with the analyte (Zhang and Fang, 2010).

Since the GCE-AONP-PANI-SWCNT modified electrode proved superior to the other electrodes investigated, its stability and resistance toward fouling effect as a result of lindane reduction product was further explored by CV using 20 scans. Figure 7B presents the CV obtained in 9 μM lindane at a scan rate of 25 mVs^−1^. Lindane reduction current was stable after 20 scans with a small current decrease (0.57 %) suggesting good stability of the GCE-AONP-PANI-SWCNT modified electrode in lindane.

### Effect of scan rate on lindane reduction

**Figure 8:**
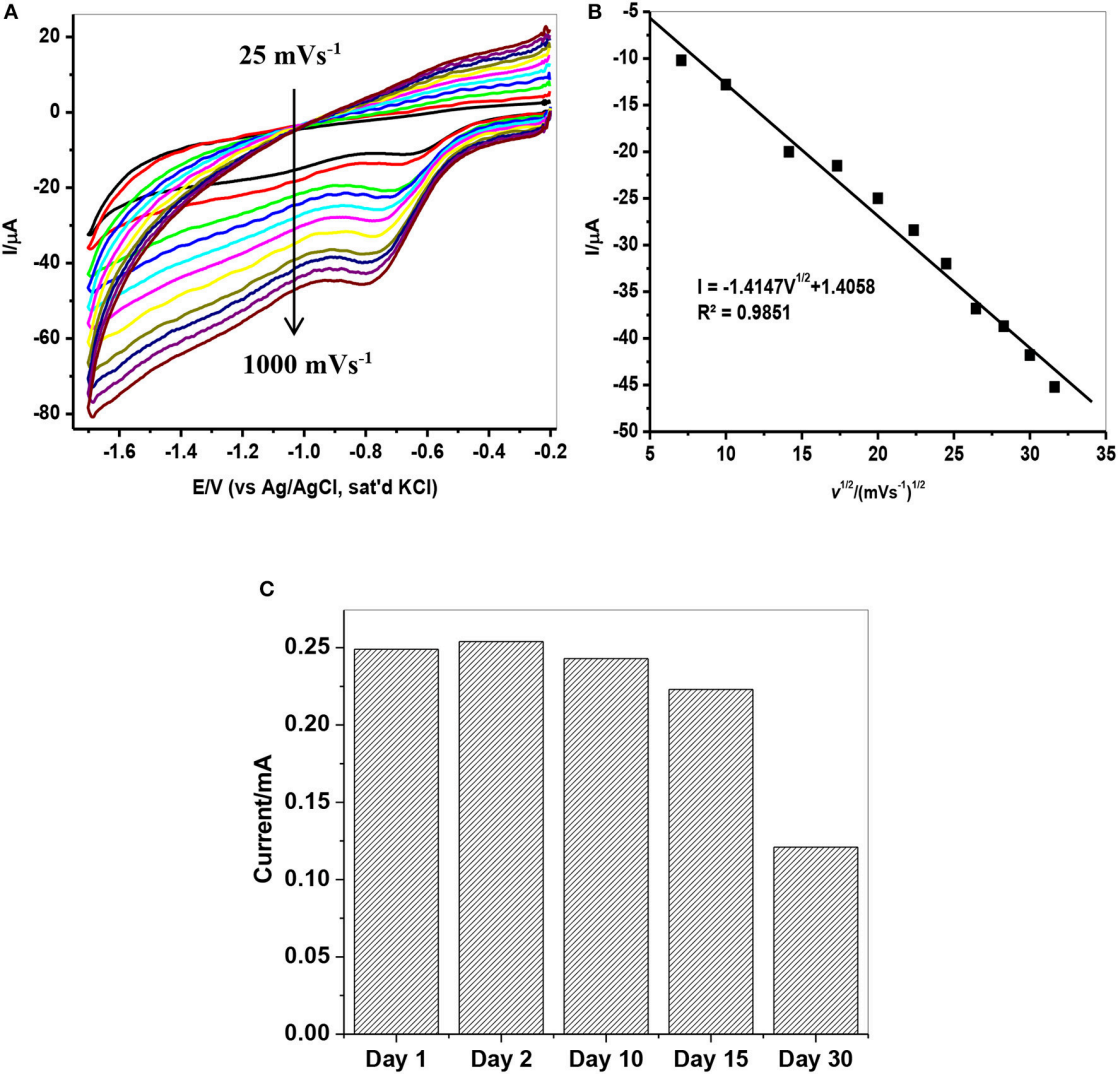
**(A)** CVs of GCE-AONP-PANI-SWCNT electrode in the presence of 9 μM lindane prepared in supporting electrolyte containing 0.05 M TBAB at a scan rate of 25–1,000 mVs^−1^. **(B)** Plot of current (I) against square root of scan rate (*v*^1/2^) for GCE-AONP-PANI-SWCNT modified electrode in lindane, and **(C)** Repeatability studies of GCE-AONP-PANI-SWCNT electrode in the presence of 9 μM for 30 successive days.

**Scheme 1:**
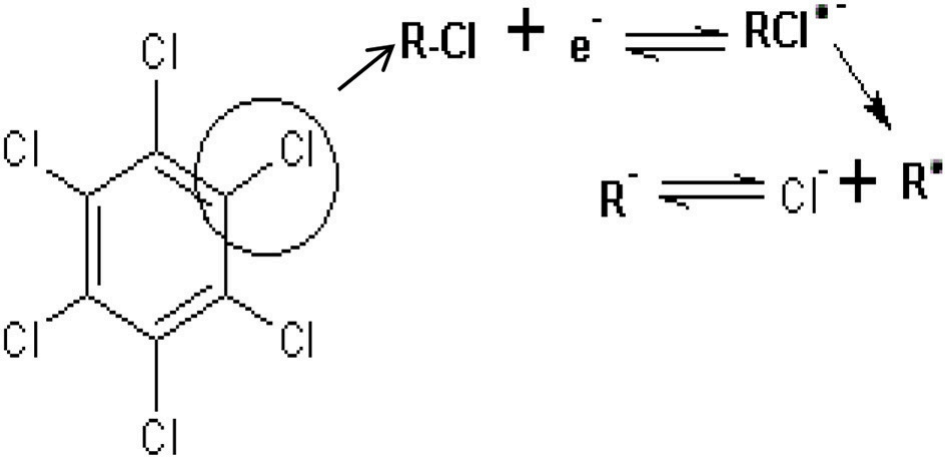
Stepwise mechanism of lindane reduction.

**Scheme 2:**
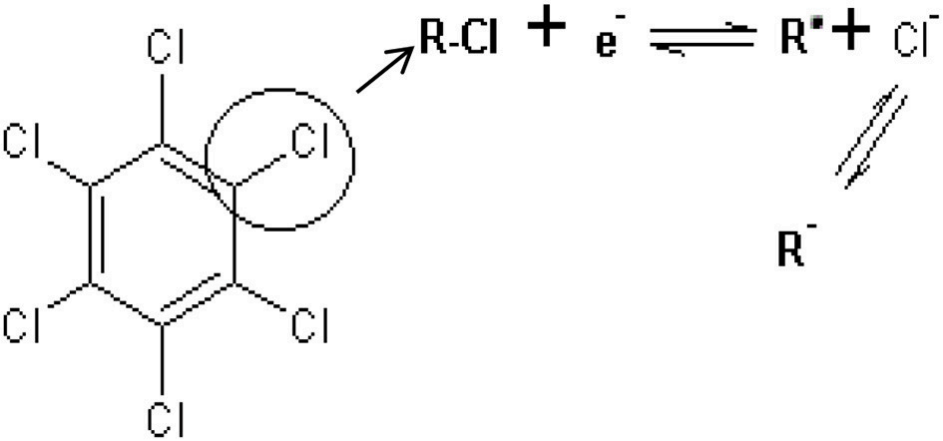
Concerted mechanism of lindane reduction.

The effect of scan rate on lindane reduction current was carried out by using CV experiments in the presence of 9 μM lindane. Figure 8A presents the cyclic voltammograms obtained for GCE-AONP-PANI-SWCNT modified electrode. It was observed that as the scan rate increases there was simultaneous increase in the peak current which suggests diffusion controlled process (Siswana et al., 2006; Hegde et al., 2008; Kumaravel et al., 2013). Figure 8B shows the plot of current (I) against square root of scan rate (*v*^1/2^) with a linear regression equation I = −1.4147*v*^1/2^ + 1.4058 and a correlation coefficient R^2^ = 0.9851. A non-zero intercept value can be attributed to process of adsorption after diffusion (Prathap et al., 2015). It was proposed that the mechanism of lindane reduction continues by dissociative electron transfer (DET) leading to the scission of the carbon-chlorine bond. The mechanism involves the formation of an intermediate radical and an anion as shown by Scheme 1, or in a concerted mechanism yielding directly a radical and an anion shown by Scheme 2 (Isse et al., 2008; Kaur et al., 2015).

The repeatability study on the modified AONP-PANI-SWCNT glassy carbon electrode was carried out in the presence of 9 μM lindane for a period of 30 successive days under the same condition. The electrode was rinsed in distilled water after every run. A 5% increase in reduction peak current was observed on the second day while after the 30th day, a drop in current by about 50% was observed as shown in Figure 8C suggesting fouling (electrode poisoning) effect. This suggests that the catalytic property of the modified electrode could only last for about 15 days with relatively little drop in current response (ca 20%). However, the composite modified electrode showed no significant drop in lindane reduction current when reused after storage in phosphate buffer solution (pH 7.0) in a refrigerator (at 4°C) for 4 weeks. Therefore, the current is reproducible.

### Electroanalysis of lindane

**Figure 9:**
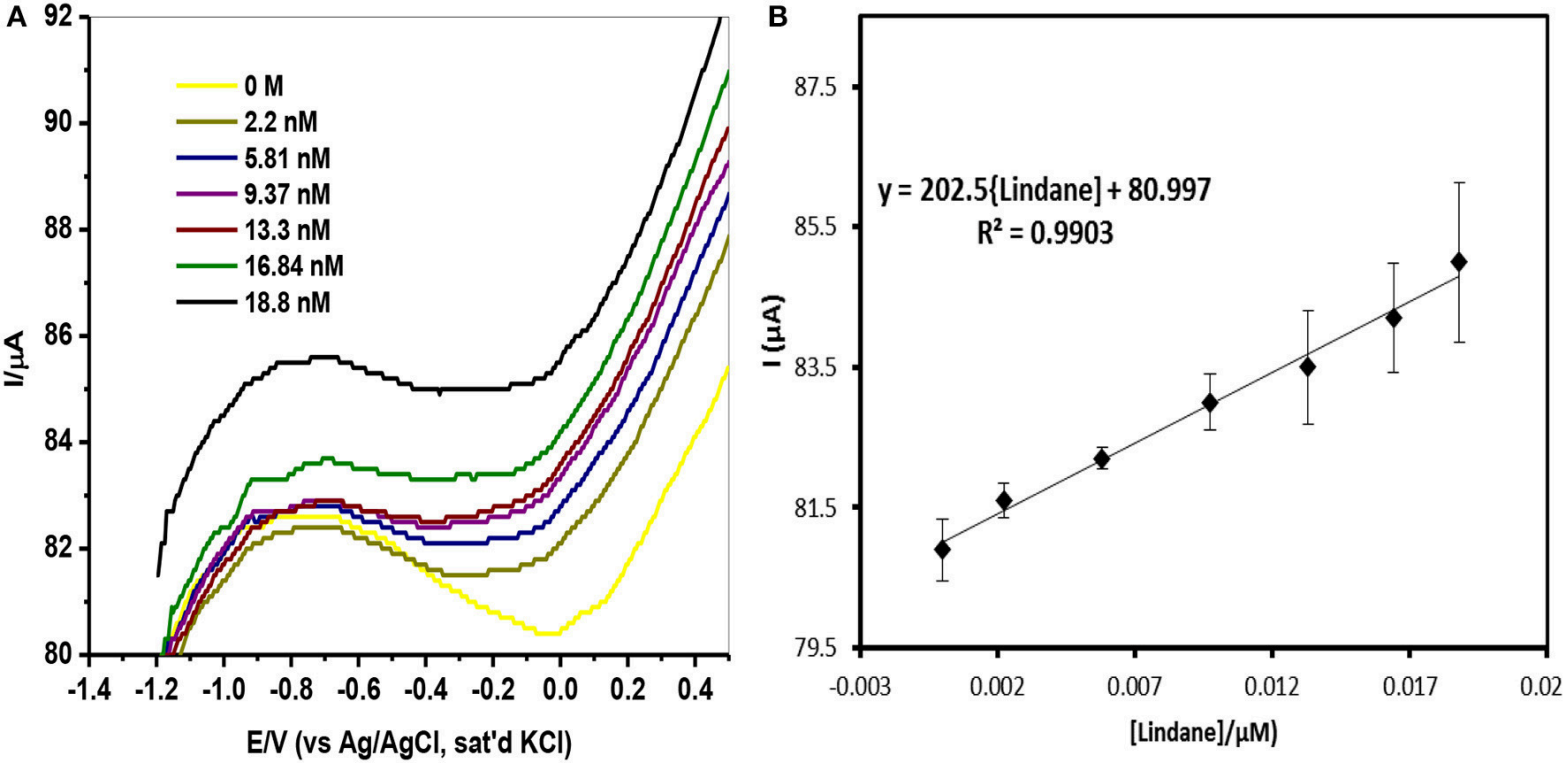
**(A)** SWV of GCE-AONP-PANI-SWCNT electrode in 0.0 nM−18.8 nM lindane. **(B)** Plot of current response versus lindane concentration and error bars represent standard deviations of three independent measurements.

Square-wave voltammetry (SWV) experiments were carried out in various concentrations of lindane prepared in 0.05 M TBAB. Figure 9A shows the square-wave voltammograms obtained for the GCE-AONP-PANI-SWCNT modified electrode with varying concentrations of lindane. Figure 9B presents plot of current response versus lindane concentration with a linear regression equation I_p_ = 202.5 [lindane] + 80.997 and correlation coefficient *R*^2^ = 0.9903. It was observed from Figure 9A that the lindane reduction peak current simultaneously increased with increase in lindane concentration and the potential gradually shifted negatively with increasing lindane concentration. In Figure 9B the linear response obtained was satisfactory over the range of lindane concentrations 0.0–18.8 nM. The standard deviation error value from the linear graph (Figure 9B) was found to be 0.21. The detection limit (LoD) for lindane at GCE-AONP-PANI-SWCNT modified electrode was calculated by using the relationship 3.3 δ/*m*, where δ is the relative standard deviation of the intercept of the y-coordinates and *m* is the slope of the same line (Adekunle et al., 2010a). The LoD, limit of quantification (LoQ) and sensitivity of the GCE-AONP-PANI-SWCNT electrode toward lindane were determined to be 2.01, 6.09 nM, and 202.5 μA/μM, respectively. The LoD and sensitivity reported here were found to be better compared to those reported at NiCo_2_O_4_ modified GCE (3.6 μM, 0.2 μA/μM) (Prathap and Srivastava, 2013), cellulose acetate modified GCE (37 μM) (Kumaravel et al., 2013), Nylon6,6/MWCNT/Fe_3_O_4_ modified GCE (32 nM) (Fayemi et al., 2016) and CuO/MnO_2_ hierarchical nano-microstructures modified electrode (4.8 nM, 0.022 μA/μM) (Prathap et al., 2015). This suggests that the AONP-PANI-SWCNT modified GCE has excellent and improved electrocatalytic activity for the electro-oxidation of lindane. Table 3 summarizes the comparison of analytical performance of AONP-PANI-SWCNT modified GCE electrode with other sensors reported for electrocatalytic detection of lindane.

**Table 3:** Comparison of analytical performance of AONP-PANI-SWCNT modified GCE electrode with other non-enzymatic sensors reported for electrocatalytic detection of lindane.

**Composite electrode**	**LoD (μM)**	**Linear range (μM)**	**Sensitivity**	**References**
NiCo_2_O_4_/GCE	3.6	10–170	0.2 μA/μM	Prathap et al., 2013
CA /GCE	37	50–1000	–	Kumaravel et al., 2013
Nylon6,6/MWCNT/Fe_3_O_4_/GCE	32 × 10^−3^	9.9–5.0	–	Fayemi et al., 2016
CuO/MnO_2_/GCE	4.8 × 10^−3^	0.0–700	0.022 μA/μM	Prathap et al., 2015
αMnO_2_-NW/GCE	114 × 10^−3^	1.1–510	–	Prathap et al., 2016
AgNPs(5%)-PANI-Nano-ZSM-5/GCE	5 × 10^−3^	0.01–90	1.24 mA/μM	Kaur et al., 2015
GCE-AONP-PANI-SWCNT	2.01 × 10^−3^	0.0 × 10^−3^ – 18.8 × 10^−3^	202.5 μA/μM	Present study

*GCE, Glassy carbon electrode; CA, Cellulose acetate; MWCNT, Multi-walled carbon nanotube; NW, Nanowires; Nano-ZSM-5, Nanocrystalline Zeolite Socony Mobil-5; PANI, Polyaniline*.

### Lindane interference studies

**Figure 10:**
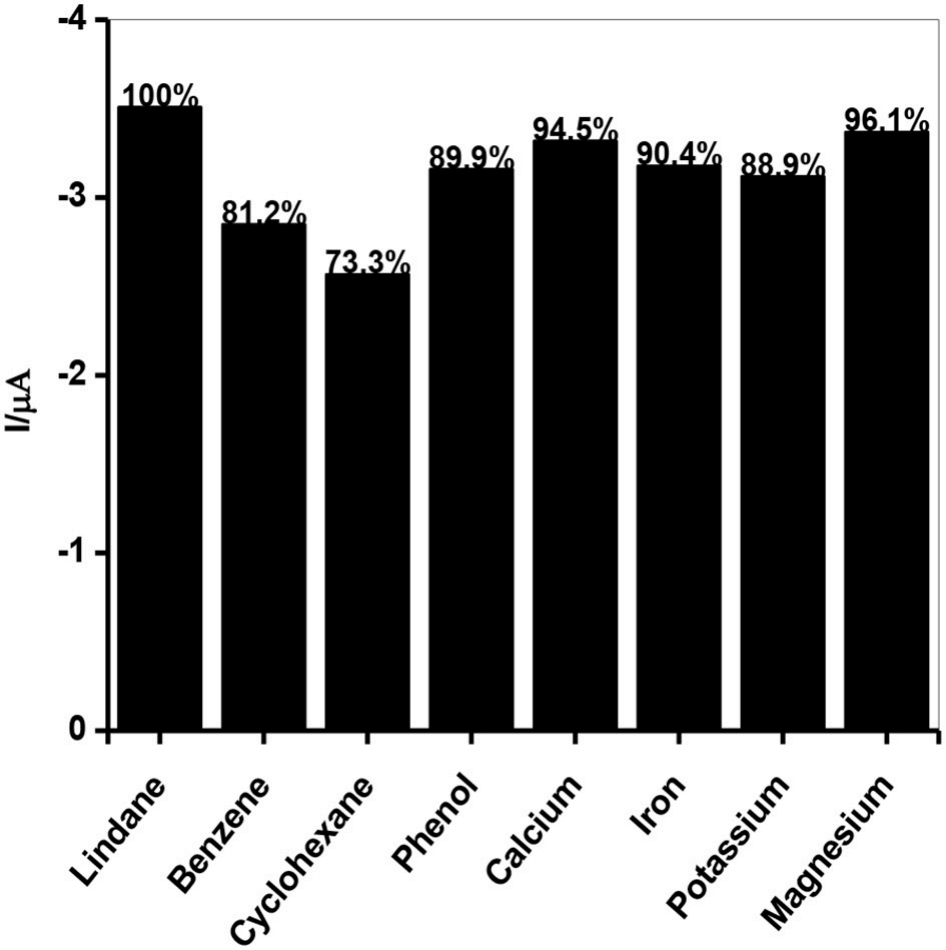
Current response of lindane reduction signal as a percentage in the presence of interfering species (benzene, cyclohexane, phenol, Ca^2+^, Fe^2+^, K^+^, and Mg^2+^) at GCE-AONP-PANI-SWCNT modified electrode by cyclic voltammetry.

Interference studies were carried out to investigate the selectivity of the developed sensor toward lindane determination using continuous chronoamperometry (CA) and CV. The selection of interfering species for this study was based on the historical use of lindane both in industry and agriculture sector and also the fact that these species may be present in water or soil from industrial areas and agricultural land (Kumaravel et al., 2013; Fayemi et al., 2016; Prathap et al., 2016). The selected species which may potentially interfere with lindane determination include electroactive organic compounds such as cyclohexane (C_6_H_12_), benzene (C_6_H_6_), phenol (C_6_H_5_OH) and some inorganic cations such as calcium (Ca^2+^), potassium (K^+^), magnesium (Mg^2+^), and iron (Fe^2+^). The chronoamperometric response at the GCE-AONP-PANI-SWCNT modified electrode with addition of 1 mM each of lindane, and the interfering species at a fixed potential of 0.2 V (graph not shown) shows that the addition of organic compounds such as C_6_H_12_, C_6_H_6_, and C_6_H_5_OH did not interfere with the current response of lindane. Presence of phenol lead to the process of hydroxylation in the reaction mixture and hence the removal or substitution of chloride from chlorobenzene (lindane) with hydroxyl ions, resulting in oxidation of lindane. Likewise, in the presence of inorganic interfering species such as Ca^2+^, K^+^, Mg^2+^, and Fe^2+^ there was no interference with lindane signal. The results obtained indicated that the GCE-AONP-PANI-SWCNT modified electrode can be used for selective determination of lindane in the presence of organic and inorganic molecules. Figure 10 shows the percentage current response of lindane reduction peak in the presence of the interfering species at GCE-AONP-PANI-SWCNT modified electrode using CV at a scan rate of 25 mVs^−1^. The results obtained showed that the GCE-AONP-PANI-SWCNT modified electrode had anti-interference behavior toward the detection of lindane in the presence of interfering species (benzene, cyclohexane, phenol, Ca^2+^, Fe^2+^, K^+^, and Mg^2+^) with an average of only 12.1% current drop on the reduction signal of lindane.

### Lindane real sample analysis

Real sample analysis was carried out to establish the practical application of the GCE-AONP-PANI-SWCNT modified electrode toward lindane detection in both river water and tap water samples. The river water samples were collected from upper crocodile sub—catchment of Crocodile River in Rustenburg of the North West province, South Africa. This river water system is known to receive runoff from agricultural land as well as effluents from the industrial mines around the area. The samples were collected from a point in the river with GPS coordinates: S 025° 40′ 09.3 E 027° 47′ 31.7. The river water was used without further purification. Lindane/water samples with concentrations ranging from 0.5–100 μM were prepared by using 60:40 methanol/river-water containing 0.05 M TBAB and CV studies were carried out to determine the concentration of lindane in the samples. Table 4 presents the results obtained and it was observed that recoveries of lindane at lower concentrations of added lindane was higher suggesting the presence of lindane in the river water samples; however at higher added concentrations of lindane recoveries were lower. This observation may be due to the sensor's kinetic range.

**Table 4:** Determination of Lindane concentration in river water and tap water samples using CV method.

**Sample**	**Compound**	**Added (μM)**	**Found^a^ (μM)**	**Recovery (%)**	**RSD (%)**
River water	Lindane	0.5	1.0	200	14.4
		1	1.6	160	10.1
		20	18.7	94	12.9
		100	100.2	100.2	0.35
Tap Water	Lindane	10	9.87	98.7	3.51
		20	20.3	101	2.99
		40	33.6	84	3.88
		50	44.3	89	4.01

a *Average value of three determinations*.

The tap water used for the study was obtained from the local municipality water supply and was used without further purification. Concentration of prepared Lindane samples added ranges from 10 to 50 μM. The sensor yielded good recoveries (99.9% average) at lower concentrations of lindane when compared to higher concentrations (86.5% average).

## Conclusions

This paper describes the successful fabrication of a sensor for the electrochemical detection of lindane. Electrocatalytic reduction of lindane was carried out using CV technique and the highest current response was recorded at the GCE-AONP-PANI-SWCNT electrode with reduction peak at −0.95 V. Stability study (20 scans) toward lindane was carried out using GCE-AONP-PANI-SWCNT electrode and the observed current drop between the 1st and 20th scan was only 0.57%. This suggested that the fabricated GCE-AONP-PANI-SWCNT sensor was stable and resistant to electrode fouling effects. The effect of scan rate was investigated using CV experiment and the plot of reduction peak currents versus square root of scan rate gave a linear relationship where reduction current increased simultaneously as the scan rate increased suggesting that lindane reduction was a diffusion controlled process. The results recorded for the LoD, LoQ, and sensitivity of the GCE-AONP-PANI-SWCNT electrode toward lindane were better compared with those reported in literature. The proposed sensor further demonstrated good selectivity and anti-interference behavior toward the detection of lindane in the presence of interfering species. Real sample analysis to establish the analytical potential of the developed GCE-AONP-PANI-SWCNT sensor was carried out using river and tap water samples. The percentage recoveries obtained at the fabricated sensor indicated its potential for the determination of lindane in river and tap water samples.

## Author contributions

EE and AA conceptualized and designed the work and were part of the manuscript write-up. KM, OF, and E-SS carried out the experiments, interpreted some of the results and were also involved in the manuscript preparation. All the authors reviewed the manuscript and have agreed to its publication.

### Conflict of interest statement

The authors declare that the research was conducted in the absence of any commercial or financial relationships that could be construed as a potential conflict of interest.

## References

[B1] Abdolahi A. Hamzah E. Ibrahim Z. Hashim S. (2012). Synthesis of uniform polyaniline nanofibers through interfacial polymerization. Materials (Basel). 5, 1487–1494. 10.3390/ma5081487

[B2] Abdollahi M. Ranjbar A. Shadnia S. Nikfar S. Rezaie A. (2004). Pesticides and oxidative stress: a review. Med. Sci. Monit. 10, RA141–RA147. 15173684

[B3] Adekunle A. S. Agboola B. O. Pillay J. Ozoemena K. I. (2010a). Electrocatalytic detection of dopamine at single-walled carbon nanotubes–iron (III) oxide nanoparticles platform. Sensor. Actuat. B Chem. 148, 93–102. 10.1016/j.snb.2010.03.088

[B4] Adekunle A. S. Ayenimo J. G. Fang X. Doherty W. O. Arotiba O. A. Mamba B. B. (2011a). Electrochemical response and impedimetric behaviour of dopamine and epinephrine at platinum electrode modified with carbon nanotubes-gold nanocomposite. Int. J. Electrochem. Sci. 2826–2844. 10.1002/elan.200804240

[B5] Adekunle A. S. Lebogang S. Gwala L. P. Tsele T. P. Olasunkanmi L. O. Fayemi O. E. (2015). Electrochemical response of nitrite and nitric oxide on graphene oxide nanoparticles doped with Prussian blue (PB) and Fe_2_O_3_ nanoparticles. RSC. Adv. 5, 27759–27774. 10.1039/C5RA02008E

[B6] Adekunle A. S. Mamba B. B. Agboola B. O. Ozoemena K. I. (2011b). Nitrite electrochemical sensor based on Prussian Blue /single-walled carbon nanotubes modified pyrolytic graphite electrode. *Int. J. Electrochem*. Sci. 6, 4388–4403.

[B7] Adekunle A. S. Pillay J. Ozoemena K. I. (2010b). Probing the electrochemical behaviourof SWCNT–cobalt nanoparticles and their electrocatalytic activities towards the detection of nitrite at acidic and physiological pH conditions. Electrochim. Acta 55, 4319–4327. 10.1016/j.electacta.2009.02.102

[B8] Anirudhan T. S. Alexander S. (2015). Design and fabrication of molecularly imprinted polymer-based potentiometric sensor from the surface modified multi-walled carbon nanotube for the determination of lindane (γ-hexachlorocyclohexane), an organochlorine pesticide. Biosens. Bioelectron. 64, 586–593. 10.1016/j.bios.2014.09.074 25310493

[B9] Bard A. J. Faulkner L. R. (2001). Electrochemical Methods: Fundamentals and Applications. New York, NY: John Wiley and Sons.

[B10] Blair A. Cantor K. P. Zahm S. H. (1998). Non-Hodgkin's lymphoma and agricultural use of the insecticide lindane. *Am. J. Ind*. Med. 33, 82–87. 10.1002/(sici)1097-0274(199801)33:1<82::aid-ajim9>3.0.co;2-y 9408531

[B11] Cesarino I. Moraes F. C. Machado S. A. S. (2011). A biosensor based on polyaniline-carbon nanotube core-shell for electrochemical detection of pesticides. Electroanalysis 23, 2586–2593. 10.1002/elan.201100161

[B12] Cesariono I. Cesarino V. Lanza M. R. V. (2013). Carbon nanotubes modified with antimony nanoparticles in a paraffin composite electrode: simultaneous determination of sulfamethoxazole and trimethoprim. Sensor. Actuat. B Chem. 188, 1293–1299. 10.1016/j.snb.2013.08.047

[B13] Chena D. Zhuanga X. Zhai J. Zhenga Y. Lua H. Chena L. (2018). Preparation of highly sensitive Pt nanoparticles-carbon quantum dots/ionic liquid functionalized graphene oxide nanocomposites and application for H_2_O_2_ detection. Sens. Actuat. B Chem. 1500–1506. 10.1016/j.snb.2017.08.156

[B14] Chiang I. W. Brinson B. E. Smalley R. E. Margrave J. L. Hauge R. H. (2001). Purification and characterization of Single-Wall Carbon Nanotubes (SWNTs) obtained from the gas-phase decomposition of CO (HiPco Process). J. Phys. Chem. B 105, 1157–1161. 10.1021/jp003453z 40999639

[B15] Chrysikou L. Gemenetzis P. Kouras A. Manoli E. Terzi E. Samara C. (2008). Distribution of persistent organic pollutants, polycyclic aromatic hydrocarbons and trace elements in soil and vegetation following a large scale landfill fire in northern Greece. Environ. Int. 34, 210–225. 10.1016/j.envint.2007.08.007 17900688

[B16] Dalui B. Basumallick I. N. Ghosh S. (2008). Zinc-poly(aniline) rechargeable battery assembled with aqueous electrolyte. Ind. J. Chem. Technol. 15, 576–580.

[B17] Deng Z. Tang F. Chen D. Meng X. Cao L. Zou B. (2006). A simple solution route to single-crystalline Sb_2_O_3_ nanowires with rectangular cross sections. J. Phys. Chem. B 110, 18225–18230. 10.1021/jp063748y 16970439

[B18] El-Shahawi M. S. Hamza A. Bashammakh A. S. Al-Saggaf W. T. (2010). An overview on the accumulation, distribution, transformations, toxicity and analytical methods for the monitoring of persistent organic pollutants. Talana 80, 1587–1597. 10.1016/j.talanta.2009.09.055 20152382

[B19] Fayemi O. E. Adekunle A. S. Ebenso E. E. (2015). Metal oxide nanoparticles/multi-walled carbon nanotube nanocomposite modified electrode for the detection of dopamine. J. Biosens. Bioelectron. 6, 190–194.

[B20] Fayemi O. E. Adekunle A. S. Ebenso E. E. (2016). A sensor for the determination of lindane using PANI/Zn, Fe(III) oxides and nylon 6,6/MWCNT/Zn, Fe(III) oxides nanofibers modified glassy carbon electrode. J. Nanomater. 2016, 78–88. 10.1155/2016/4049730

[B21] Fu C. Yang W. Chen X. Evans G. D. (2009). Direct electrochemistry of glucose oxidase on a graphite nanosheet–nafion composite film modified electrode. Electrochem. Commun. 997–1000. 10.1016/j.elecom.2009.02.042

[B22] Gill S. Bowers W. J. Nakai J. S. Yagminas A. Mueller R. Pulido O. (2013). EffectsOf environmentally relevant mixtures of persistent organic pollutants on the developmental neurobiology in rats. Toxicol. Pathol. 41, 38–47. 10.1177/0192623312451370 22872703

[B23] Hegde R. N. Swamy B. E. K. Sherigara B. S. Nandibewoor S. T. (2008). Electro-oxidation of atenolol at a glassy carbon electrode. *Int. J. Electrochem*. Sci. 3, 302–314.

[B24] Helali S. Bohli N. Mostafa H. M. A. Zina H. B. Al-Hartomy O. A. Abdelghani A. (2016). Electrical impedance spectroscopy using single wall carbon nanotubes carboxlic acid functionalized: detection of copper in Tabuk-Kingdom of Saudi Arabia water. J. Nanomed. Nanotechnol. 7:396 10.4172/2157-7439.1000396

[B25] Isse A. A. Gottardelo S. Durante C. Gennaro A. (2008). Dissociative electron transfer to organic chlorides: electrocatalysis at metal cathodes. Phys. Chem. Chem. Phys. 10, 2409–2416. 10.1039/b719936h 18414732

[B26] Jones K. C. de Voogt P. (1999). Persistent organic pollutants (POPs): state of the science. Environ. Pollut. 100, 209–221. 10.1016/S0269-7491(99)00098-6 15093119

[B27] Kaur B. Srivastara R. Satpati B. (2015). Silver nanoparticle decorated polyaniline–zeolite nanocomposite material based non-enzymatic electrochemical sensor for nanomolar detection of lindane. RSC Adv. 5, 57657–57665. 10.1039/C5RA09461E

[B28] Kavitha B. Kurma K. S. Narsimlu N. (2013). Synthesis and characterization of polyaniline nano-fibers. Ind. J. Pure Appl. Phys. 51, 207–209.

[B29] Kaviyarasu K. Sajan D. Devarajan P. A. (2013). A rapid and versatile method forsolvothermal synthesis of Sb_2_O_3_ nanocrystals under mild conditions. Appl. Nanosci. 3, 529–533. 10.1007/s13204-012-0156-y

[B30] Khalef W. K. (2013). Synthesis of antimony oxide nanoparticles by pulsed laser ablationin wet media. Iraqi J. Appl. Phy. 9, 5–13.

[B31] Konyushenko E. N. Reynard S. Pellerin V. Trchová M. Stejskal J. Supurina I. (2011). Polyaniline prepared in ethylene glycol or glycerol. Polymer 52, 1900–1907. 10.1016/j.polymer.2011.02.047

[B32] Kumaravel A. Vincent S. Chandrasekaran S. (2013). Development of an electroanalytical sensor for α-hexachlorocyclohexane based on a cellulose acetate modified glassy carbon electrode. Anal. Methods 5, 931–938. 10.1039/c2ay26119g

[B33] Lichtenstein E. P. Beck S. D. Schulz K. R. (1956). Colorimetric determination of lindane in soils and crops. J. Agric. Food Chem. 4, 936–944. 10.1021/jf60069a002

[B34] Lisdat F. Shäfer D. (2008). The use of electrochemical impedance spectroscopy forbiosensing. Anal. Bioanal. Chem. 391, 1555–1567. 10.1007/s00216-008-1970-7 18414837

[B35] Liu G. Wang S. Liu J. Song D. (2012). An electrochemical immunosensor based onchemical assembly of vertically aligned carbon nanotubes on carbon substrates for direct detection of the pesticide endosulfan in environmental water. Anal. Chem. 84, 3921–3928. 10.1021/ac202754p 22448910

[B36] Naidu B. S. Pandey M. Sudarsan V. Vatsa R. K. Tewari R. (2009). Photoluminescence and Raman spectroscopic investigations of morphology assisted effects in Sb_2_O_3_. Chem. Phys. Lett. 474, 180–184. 10.1016/j.cplett.2009.04.050

[B37] Ng S. H. Wang J. Guo Z. P. Chen J. Wang G. X. Liu H. K. (2005). Single wall carbon nanotube paper as anode for lithium-ion battery. *Electrochim*. Acta 51, 23–28. 10.1016/j.electacta.2005.04.045

[B38] Norén K. Westöö G. (1968). Determination of some chlorinated pesticides invegetable oils, margarine, butter, milk, eggs, meat, and fish by gas chromatography and thin-layer chromatography. Acta Chem. Scand. 22, 2289–2293. 10.3891/acta.chem.scand.22-2289 4179951

[B39] Olad A. Ilghami F. Nosrati R. (2012). Surfactant-assisted synthesis of polyaniline nanofibers without shaking and stirring: effect of conditions on morphology and conductivity. Chem. Papers 66, 757–764. 10.2478/s11696-012-0197-4

[B40] Otles S. (2016). Handbook of Food Analysis Instruments. New York, NY: CRC Press.

[B41] Peng H. Alemang L. B. Margrave J. L. Khabasheski V. N. (2003). Sidewall carboxylic acid functionalization of single-walled carbon nanotubes. J. AM.Chem. Soc. 125, 15174–15182. 10.1021/ja037746s 14653752

[B42] Prathap A. M. U. Sun S. Wei C. Xu Z. J. (2015). A novel non-enzymatic lindanesensor based on CuO–MnO_2_ hierarchical nano-microstructures for enhanced sensitivity. Chem. Commun. 51, 4376–4379. 10.1039/C5CC00024F 25674914

[B43] Prathap A. M. U. Sun S. Xu Z. J. (2016). An electrochemical sensor highly selective forlindane determination: a comparative study using three different α-MnO_2_ nanostructures. RCS Adv. 6, 22973–22979. 10.1039/C5RA26771D

[B44] Prathap M. U. A. Srivasta R. (2011). Morphological controlled synthesis of micro-/nano-polyaniline. J. Polym. Res. 18, 2455–2467. 10.1007/s10965-011-9662-y

[B45] Prathap M. U. A. Srivastava R. (2013). Electrochemical reduction of lindane (γ-HCH) atNiCo_2_O_4_ modified electrode. Electrochim. Acta 108, 145–152. 10.1016/j.electacta.2013.06.122

[B46] Prathap M. U. A. Srivastava R. Satpati B. (2013). Simultaneous detection of guanine,adenine, thymine, and cytosine at polyaniline/MnO_2_ modified electrode. Electrochim. Acta 114, 285–294. 10.1016/j.electacta.2013.10.064

[B47] Price B. K. Tour J. M. (2006). Functionalization of single-walled carbon nanotubes “On Water.” J. Am. Chem. Soc. 128, 12899–12904. 10.1021/ja063609u 17002385

[B48] Rahman M. A. Kumar P. Park D. S. Shim Y. B. (2008). Electrochemical sensorsbased on organic conjugated polymers. Sensors 8, 118–141. 10.3390/s8010118 27879698 PMC3681146

[B49] Rodrigues M. V. N. Reyes F. G. R. Magalhaes P. M. Rath S. (2007). GC-MS determination of organochlorine pesticides in medicinal plants harvested in Brazil. J. Braz. Chem. Soc. 18, 135–142. 10.1590/S0103-50532007000100015

[B50] Sairam M. Nataraj S. K. Aminabhavi T. M. Roy S. Madhusoodana C. D. (2006). Polyaniline membranes for separation and purification of gases, liquids, and electrolyte solutions. Sep. Purif. Rev. 35, 249–283. 10.1080/15422110600859727

[B51] Salihovic S. Mattioli L. Lindstrom G. Lind L. Lind P. M. Van Bavel B. (2012). A rapid method for screening of the Stockholm convention POPs in small amounts of human plasma using SPE and HRGC/HRMS. Chemosphere 86, 747–753. 10.1016/j.chemosphere.2011.11.006 22153485

[B52] Sen I. Shandil A. Aggarwal M. Khandal R. K. (2011). Simultaneous determinationby gas chromatography of lindane and carbaryl in combined formulations. E J. Chem. 8, 391–399. 10.1155/2011/195495

[B53] Shap M. Petersson M. Edstrom K. (1979). Preliminary determinations of electron transfer kinetics involving ferrocene covalently attached to a platinum surface. J. Electroanal Chem. 95, 123–130.

[B54] Silwana B. van der Horst C. Iwuoha E. Somerset V. (2015). Synthesis, characterisation and electrochemical evaluation of reduced graphene oxide modified antimony nanoparticles. Thin Solid Films 592, 124–134. 10.1016/j.tsf.2015.09.010

[B55] Siswana M. Ozoemena K. I. Nyokong T. S. (2006). Electrocatalytic behaviour ofcarbon paste electrode modified with iron(II) phthalocyanine (FePc) nanoparticles towards the detection of amitrole. Talanta 69, 1136–1142. 10.1016/j.talanta.2005.12.014 18970694

[B56] Tchoul M. N. Ford W. T. Lollo G. Resasco D. E. Arepalli S. (2007). Effect of mild nitric acid oxidation on dispersability, size, and structure of single-walled carbon nanotubes. Chem. Mater. 19, 5765–5772. 10.1021/cm071758l

[B57] Tovide O. Jaheed N. Mohamed N. Nxusani E. Sunday C. Tsegaye A. (2014). Graphenated polyaniline-doped tungsten oxide nanocomposite sensor for real time determination of phenanthrene. Electrochim. Acta 128, 138–148. 10.1016/j.electacta.2013.12.134

[B58] Trchová M. Stejskal J. (2011). Polyaniline: the infrared spectroscopy of conducting polymer nanotubes (IUPAC Technical Report). Pure Appl. Chem. 83, 1803–1817. 10.1351/PAC-REP-10-02-01

[B59] Vairavapandian D. Vichulada P. Lay M. D. (2008). Preparation and modification ofcarbon nanotubes: review of recent advances and applications in catalysis and sensing. Anal. Chem. Acta 626, 119–129. 10.1016/j.aca.2008.07.052 18790113

[B60] Van Dyk J. S. Pletschke B. (2011). Review on the use of enzymes for the detection of organochlorine, organophosphate and carbamate pesticides in the environment. Chemosphere 82, 291–307. 10.1016/j.chemosphere.2010.10.033 21055790

[B61] Vassilakis I. Tsipi D. Scoulos M. (1998). Determination of a variety of chemicalclasses of pesticides in surface and ground waters by off-line solid-phase extraction, gas chromatography with electron-capture and nitrogen–phosphorus detection, and high-performance liquid chromatography with post-column derivatization and fluorescence detection. J. Chromatogr. A 823, 49–58. 10.1016/S0021-9673(98)00181-2 9818392

[B62] Wilson C. Tisdell C. (2001). Why farmers continue to use pesticides despite environmental, health and sustainability costs. Ecol. Econ. 39, 449–462. 10.1016/S0921-8009(01)00238-5

[B63] Xu K. Lin W. Wu J. Peng J. Xing Y. Gao S. (2015). Construction and electronic properties of carbon nanotube hybrids with conjugated cubic silsesquioxane. New J. Chem. 39, 8405–8415. 10.1039/C5NJ01376C

[B64] Yu Q. Z. Shi M. M. Deng M. Wang M. Chen H. Z. (2008). Morphology and conductivity of polyaniline sub-micron fibers prepared by electrospinning. Mat. Sci. Eng. B Solid. 150, 69–70. 10.1016/j.mseb.2008.02.008

[B65] Zhang L. Fang M. (2010). Nanomaterials in pollution trace detection and environmental improvement. Nanotoday 5, 128–142. 10.1016/j.nantod.2010.03.002

[B66] Zhang L. Su K. Li X. (2002). Electrorheological effects of polyaniline-typeelectrorheological fluids. J. Appl. Polym. Sci. 87, 733–740. 10.1002/app.11356

[B67] Zhang Z. Guo L. Wang W. (2001). Synthesis and characterization of antimony oxide nanoparticles. J. Mater. Res. 16, 803–805. 10.1557/JMR.2001.0096

[B68] Zhuang X. Tian C. Luan F. Wu X. Chen L. (2016). One-step electrochemical fabrication of a nickel oxide nanoparticle/polyaniline nanowire/graphene oxide hybrid on a glassy carbon electrode for use as a non-enzymatic glucose biosensor. RSC Adv. 92541–92546. 10.1039/C6RA14970G

[B69] Zhuanga X. Chena D. Wanga S. Liua H. Chena L. (2017). Manganese dioxide nanosheet-decorated ionic liquid-functionalized graphene for electrochemical theophylline biosensing. Sens. Actuat. B 185–191. 10.1016/j.snb.2017.05.049

